# FLC-mediated flowering repression is positively regulated by sumoylation

**DOI:** 10.1093/jxb/ert383

**Published:** 2013-11-11

**Authors:** Ga Hyun Son, Bong Soo Park, Jong Tae Song, Hak Soo Seo

**Affiliations:** ^1^Department of Plant Science, Research Institute for Agriculture and Life Sciences, and Plant Genomics and Breeding Institute, Seoul National University, Seoul 151-921, Korea; ^2^School of Applied Biosciences, Kyungpook National University, Daegu 702-701, Korea; ^3^Bio-MAX Institute, Seoul National University, Seoul 151-818, Korea

**Keywords:** AtSIZ1, FLC, flowering, post-translational modification, SUMO, sumoylation.

## Abstract

Flowering locus C (FLC), a floral repressor, is a critical factor for the transition from the vegetative to the reproductive phase. Here, the mechanisms regulating the activity and stability of the FLC protein were investigated. Bimolecular fluorescence complementation and *in vitro* pull-down analyses showed that FLC interacts with the E3 small ubiquitin-like modifier (SUMO) ligase AtSIZ1, suggesting that AtSIZ1 is an E3 SUMO ligase for FLC. *In vitro* sumoylation assays showed that FLC is modified by SUMO in the presence of SUMO-activating enzyme E1 and conjugating enzyme E2, but its sumoylation is inhibited by AtSIZ1. In transgenic plants, inducible *AtSIZ1* overexpression led to an increase in the concentration of FLC and delayed the post-translational decay of FLC, indicating that AtSIZ1 stabilizes FLC through direct binding. Also, the flowering time in mutant *FLC* (K154R, a mutation of the sumoylation site)-overexpressing plants was comparable with that in the wild type, whereas flowering was considerably delayed in *FLC*-overexpressing plants, supporting the notion that sumoylation is an important mechanism for FLC function. The data indicate that the sumoylation of FLC is critical for its role in the control of flowering time and that AtSIZ1 positively regulates FLC-mediated floral suppression.

## Abstract (precis)

Sumoylation is critical modification for protein function and stability. Floral transition activity of FLOWERING LOCUS C (FLC), a central flowering switch, is increased by sumoylation. E3 SUMO ligase SIZ1 stabilizes FLC, which results in positive regulation of FLC-mediated floral suppression

## Introduction

In eukaryotic cells, protein function and stability are post-translationally regulated by small and large molecules such as phosphates, carbohydrates, lipids, and small proteins (Castro *et al.*, 2012). The post-translational modification of target proteins by small ubiquitin-like modifier (SUMO) is an important regulatory mechanism (Wilkinson *et al.*, 2010). The reversible covalent attachment of SUMO to a lysine residue in a target protein is catalysed by E3 SUMO ligases, although conjugation of SUMO to target proteins can occur without the help of an E3 SUMO ligase (Wilkinson *et al.*, 2010). As in other eukaryotes, SUMO modification in plants has been implicated in numerous basic cellular processes, such as stress and defence responses, nitrogen metabolism, and the regulation of flowering (Hotson *et al.*, 2003; Kurepa *et al.*, 2003; Lois *et al.*, 2003; Murtas *et al.*, 2003; Miura *et al.*, 2005, 2007; Catala *et al.*, 2007; Lee *et al.*, 2007; Conti *et al.*, 2008; Yoo *et al.*, 2006; Park *et al.*, 2011).

AtSIZ1, a Siz/PIAS (SP)-RING-finger protein, regulates plant responses to nutrient deficiency and environmental stresses, and controls vegetative growth and development (Miura *et al.*, 2005, 2007, 2010; Catala *et al.*, 2007; Lee *et al.*, 2007; Yoo *et al.*, 2006; Park *et al.*, 2011; Garcia-Dominguez *et al.*, 2008; Jin *et al.*, 2008; Miura and Ohta, 2010). Due to its important roles in a wide range of physiological processes, sumoylation has been the subject of a growing number of studies in the past decades. Recently, two separate studies have identified a significant number of SUMO conjugates using proteomics methods and yeast two-hybrid screening in *Arabidopsis* under non-stress and stress conditions (Elrouby and Coupland, 2010; Miller *et al.*, 2010). The results indicate that sumoylation can regulate diverse biological processes, although the functional consequences of this modification have not been fully characterized. Only a few *Arabidopsis* proteins, such as the nitrate reductases NIA1 and NIA2, inducer of CBF expression 1 (ICE1), the R2R3-type transcription factor MYB30, and the SUMO machinery proteins AtSIZ1 and AtSCE1, have been experimentally demonstrated to be sumoylated (Miura and Hasegawa, 2010; Park *et al.*, 2011; Zheng *et al.*, 2012).

Flowering time is a critical trait in higher plants, as the timing of the transition from the vegetative to the reproductive phase is essential for reproductive success. Several genes are involved in floral induction in *Arabidopsis*, among which that encoding the MADS-box transcription factor flowering locus C (*FLC*) plays an important role in phase transition (Samach *et al.*, 2000; Simpson and Dean, 2002). The expression of *FLC* is negatively regulated by vernalization and by components of the autonomous pathway (Michaels and Amasino, 1999; Sheldon *et al.*, 1999). Vernalization-induced histone modifications are mediated by VRN1, VRN2, VRN5, and VIN3 (He and Amasino, 2005; Greb *et al.*, 2007), leading to the repression of *FLC* expression. In addition, FVE, FLD, AtSWP1, and AtCZS, which participate in the autonomous pathway, modulate the histone deacetylation of FLC chromatin (He and Amasino, 2005; Krichevsky *et al.*, 2006), repressing the transcription of the *FLC* gene. *FLC* transcription is also repressed by RNA-binding or processing proteins such as FCA, FY, FPA, FLK, and LD (Michaels and Amasino, 1999). Two recent reports have shown that FLC transcription is tightly controlled by long non-coding RNAs such as COOLAIR and COLDAIR, although their regulatory roles differ (Swiezewski *et al.*, 2009; Heo and Sung, 2011). In addition, *FLC* transcription is positively regulated by FRI and EFS, an *Arabidopsis* PAF1 homologue (He and Amasino, 2005; Kim *et al.*, 2005; Zhao *et al.*, 2005). Although several factors affecting the transcription of *FLC* have been described, the post-translational regulation of FLC stability and function has not been clearly characterized.

A recent study has shown that FLC is polyubiquitinated by SINAT5 *in vitro* (Park *et al.*, 2007), indicating that its stability may be regulated by a specific E3 ubiquitin ligase. This result suggests that the regulation of the floral transition by FLC involves a post-translational mechanism.

In the present study, it is shown that sumoylation plays a role in the regulation of flowering time by modulating the activity of FLC. AtSIZ1 stabilizes FLC through direct interaction, and it inhibits FLC sumoylation *in vitro*. Overexpression of *mFLC*, a sumoylation site mutant gene, had no effect on flowering time. These findings indicate that FLC is stabilized by the E3 SUMO ligase AtSIZ1, and FLC-mediated flowering repression is stimulated by sumoylation.

## Materials and methods

### Plant materials and growth conditions

The wild-type *Arabidopsis thaliana* plants used in this study were of the Columbia-0 (Col-0) ecotype. For plants grown in medium, seeds were surface-sterilized in commercial bleach that contained 5% sodium hypochlorite and 0.1% Triton X-100 solution for 10min, rinsed five times in sterilized water, and stratified at 4 °C for 2 d in the dark. Seeds were planted on agar plates containing Murashige and Skoog (MS) medium, 2% sucrose, and 0.8% agar, buffered to pH 5.7. For plants grown in soil, seeds were directly sown into sterile vermiculite. All plants including seedlings were grown at 22 °C under a 16h light/8h dark cycle in a growth chamber.

### Construction of recombinant plasmids

To produce His_6_-FLC, the cDNA encoding full-length FLC was amplified by PCR and inserted into the pET28a vector (Novagen). To produce glutathione *S*-transferase (GST)–AtSIZ1 or its deletion mutants, the cDNAs encoding either the full length or the deletion mutants of AtSIZ1 cDNA were inserted into the pGEX4T-1 vector (Amersham Biosciences). GST–AtSIZ1 (D1), GST–AtSIZ1 (D2), and GST–AtSIZ1 (D3) contained amino acids 90–470, 300–470, and 1–100 of AtSIZ1, respectively. For the maltose-binding protein (MBP)–AtSIZ1-haemagglutinin (HA) fusion, a cDNA encoding full-length AtSIZ1 was amplified by PCR using a primer tagged with HA and inserted into the pMALc2 vector (New England Biolabs).

For His_6_-FLC-Myc and GST–FLC-Myc production, cDNA encoding full-length FLC was amplified by PCR using primers tagged with Myc and inserted into pET28a and pGEX4T-1, respectively.

To produce the FLC mutant proteins GST–FLC(K5R)-Myc, GST–FLC(K135R)-Myc, GST–FLC(K154R)-Myc, and His_6_-FLC(K154R)-Myc (the numbers indicate the positions of the lysines in FLC that were mutated to arginine), GST–FLC-Myc and His_6_-FLC-Myc were subjected to site-directed mutagenesis using overlapping primers (Supplementary Table S1 available at *JXB* online). The double mutants GST–FLCm1(K5R, K135R)-Myc, GST–FLCm2(K5R, K154R)-Myc, and GST–FLCm3(K135R, K154R)-Myc were also generated by site-directed mutagenesis of GST–FLC(K5R)-Myc, GST-FLC(K135R)–Myc, and GST–FLC(K154R)-Myc using overlapping primers (Supplementary Table S1).

The *Arabidopsis* SUMO1 full-length cDNA was amplified by PCR with gene-specific primers and inserted into pET28a to produce the His_6_-AtSUMO1-GG, containing full-length FLC extended with GG at the 3’ end. To produce GST–IAA4 (INDOLEACETIC ACID 4), the cDNA encoding full-length IAA4 was amplified by PCR with gene-specific primers and inserted into the pGEX4T-1 vector.

*Arabidopsis* SUMO E1 and E2 enzyme-encoding constructs were kindly provided by Dr H.-P. Stuible (Colby *et al.*, 2006).

All constructs were transformed into *Escherichia coli* BL21/DE3 (pLysS) cells. The transformed cells were treated with IPTG (isopropyl-β-d-thiogalactoside) to induce fusion protein expression.

The sequences of the primers used in this study are listed in Supplementary Table S1 at *JXB* online. All the constructs were verified by automatic DNA sequencing to ensure that no mutations were introduced

### Production of transgenic *Arabidopsis* plants

To produce FLC- or mFLC (K154R)-overexpressing plants, the corresponding full-length cDNAs were amplified by PCR using a forward primer and a reverser primer tagged with FLAG_3_ and inserted into the plant expression vector pBA002. Recombinant plasmids *35S-FLC-FLAG*_*3*_ and *35S-mFLC-FLAG*_*3*_ were introduced into *Arabidopsis* by floral dipping (Clough and Bent, 1998). To produce double transgenic plants, the full-length cDNA encoding AtSIZ1 was amplified by PCR using a forward primer tagged with HA_3_ and a reverser primer and inserted into the plant expression vector pER8. The resulting recombinant plasmids *XVE-HA*_*3*_ *-AtSIZ1* and *35S-FLC-FLAG*_*3*_ were also introduced into *Arabidopsis* by floral dipping.

### Purification of recombinant proteins

All of the recombinant proteins were expressed in *E. coli* strain BL21 and were purified in accordance with the manufacturer’s instructions. Briefly, for His_6_-AtSAE1b, His_6_-AtSAE2, His_6_-AtSCE1, His_6_-AtSUMO1, His_6_-FLC, His_6_-mFLC, His_6_-FLC-Myc, and His_6_-mFLC-Myc purification, bacteria were lysed in 50mM NaH_2_PO_4_ (pH 8.0), 300mM NaCl, 1% Triton X-100, 1mM imidazole, 5mM dithiothreitol (DTT), 2mM phenylmethylsulphonyl fluoride (PMSF), and a proteinase inhibitor cocktail (Roche), and purified on Ni^2+^-nitrilotriacetate (Ni^2+^-NTA) resins (Qiagen). For GST, GST–AtSIZ1 (GS), GST–AtSIZ1 (D1), GST–AtSIZ1 (D2), GST–AtSIZ1 (D3), GST–AtSUMO1, GST–FLC-Myc, GST–mFLC-Myc, GST–FLCm1-Myc, GST–FLCm2-Myc, GST–FLCm3-Myc, and GST–IAA4 purification, bacteria were lysed in PBS buffer (pH 7.5) containing 1% Triton X-100, 2mM PMSF, and a proteinase inhibitor cocktail (Roche), and purified on glutathione resins (Pharmacia). For MBP–AtSIZ1 purification, bacteria were lysed in 20mM TRIS-HCl (pH 7.4), 200mM NaCl, 1mM EDTA, 1% Triton X-100, and 2mM PMSF containing a proteinase inhibitor cocktail (Roche), and purified on amylose resins (New England Biolabs). Protein concentrations were determined by the Bradford assay (Bio-Rad). For MBP–AtSIZ1-HA, bacteria were lysed in 50mM TRIS-HCl pH 7.5, 200mM NaCl, 1% Triton X-100, 5mM dithiothreitol (DTT), 2mM PMSF, and a proteinase inhibitor cocktail (Roche), and purified on amylose resins (New England Biolabs).

### *In vitro* binding assay

To examine the *in vitro* binding of GST–AtSIZ1 to His_6_-FLC, 2 μg of full-length GST–AtSIZ1 or deletion mutant baits and 2 μg of full-length His_6_-FLC prey were added to 1ml of binding buffer [50mM TRIS-HCl (pH 7.5), 100mM NaCl, 1% Triton X-100, 0.2% glycerol, 0.5mM β-mercaptoethanol]. After incubation at 25 °C for 2h, the reaction mixtures were incubated with a glutathione resin for 2h before washing six times with buffer [50mM TRIS-HCl (pH 7.5), 100mM NaCl, 1% Triton X-100]. Absorbed proteins were analysed by 11% SDS–PAGE and detected by western blotting using an anti-His antibody (Santa Cruz Biotechnology).

To examine the dimerization of the FLC protein, 2 μg of full-length GST–FLC bait and 2 μg of full-length His_6_-FLC or His_6_-mFLC prey were added to 1ml of binding buffer as described above. After incubation at 25 °C for 2h, the reaction mixtures were incubated with a glutathione resin and absorbed proteins were analysed as described above.

For determination of the *in vitro* binding of the FLC mutant protein His_6_-mFLC to MBP–AtSIZ1, 2 μg of full-length MBP–AtSIZ1 bait and 2 μg of full-length His_6_-FLC or His_6_-mFLC prey were added to 1ml of binding buffer as described above. After incubation at 25 °C for 2h, the reaction mixtures were incubated with an amylose resin for 2h before washing six times with buffer [50mM TRIS-HCl (pH 7.5), 100mM NaCl, 1% Triton X-100]. Absorbed proteins were analysed as described above.

### Sumoylation assays

*In vitro* sumoylation was performed in 30 μl of reaction buffer [200mM HEPES (pH 7.5), 5mM MgCl_2_, 2mM ATP] with 50ng of His_6_-AtSAE1b, 50ng of His_6_-AtSAE2, 50ng of His_6_-AtSCE1, 8 μg of His_6_-AtSUMO1-GG, and 100ng of His_6_-FLC-Myc (or GST–FLC-Myc) with or without 500ng of MBP–AtSIZ1-HA. After incubation for 3h at 30 °C, the reaction mixtures were separated on 10% SDS–polyacrylamide gels. Sumoylated His_6_-FLC-Myc or GST–FLC-Myc was detected by western blotting using an anti-Myc antibody (Santa Cruz Biotechnology).

To identify the sumoylation site on FLC, GST–FLCm1-Myc, GST–FLCm2-Myc, GST–FLCm3-Myc, and GST–mFLC-Myc were added to the reaction mixtures instead of His_6_-FLC-Myc or GST–FLC-Myc, respectively. The reaction and the subsequent steps were as described above.

To confirm the identity of the sumoylated FLC band, the sumoylation reaction was performed with GST–AtSUMO1-GG instead of His_6_-AtSUMO1-GG under the reaction conditions described above.

### Bimolecular fluorescence complementation of AtSIZ1 and FLC

To generate constructs for the bimolecular fluorescence complementation (BiFC) protein interaction assay, the cDNAs for AtSIZ1 and FLC were cloned into the pDONR201 vector. Next, the cDNAs for AtSIZ1 and FLC were transferred from their respective entry clones to the gateway vector pSAT4-DEST-n(174)EYFP-C1 (ABRC stock number CD3-1089) or pSAT5-DEST-c(175-end)EYFP-C1(B) (ABRC stock number CD3-1097), which contained the N-terminal 174 amino acids of enhanced yellow fluorescent protein (EYFP^N^) or the C-terminal 64 amino acids of EYFP (EYFP^C^). The fusion constructs encoding nEYFP–SIZ1 and cEYFP–FLC proteins were mixed at a 1:1 ratio and co-bombarded into onion epidermal cells using a helium biolistic gun. Bombarded tissues were incubated at 25 °C in the dark for 16h and YFP signals were observed by confocal laser scanning microscopy.

### Effects of AtSIZ1 overexpression on FLC concentration *in vivo*

Fourteen-day-old light-grown (16h light/8h dark) plants carrying *35S-FLC-FLAG*_*3*_ and *XVE-HA*_*3*_ *-AtSIZ1* or *35S-mFLC-FLAG*_*3*_ and *XVE-HA*_*3*_ *-AtSIZ1* transgenes on MS medium were treated in the light with or without β-oestradiol for 15h. Samples were ground in liquid nitrogen and lysates were separated by SDS–PAGE. FLC–FLAG_3_ and mFLC–FLAG_3_ levels were examined by western blotting with anti-FLAG antibody. HA_3_-AtSIZ1 induction was analysed by western blotting with anti-HA antibody. Post-translational degradation of FLC was examined using double transgenic plants of *35S-FLC-FLAG*_*3*_ and *XVE-HA*_*3*_ *-AtSIZ1* or *35S-mFLC-FLAG*_*3*_ and *XVE-HA*_*3*_ *-AtSIZ1*. Transgenic plants were incubated in liquid medium with β-oestradiol for 15h for the induction of AtSIZ1 expression, washed, and then transferred to MS medium with 100 μM cycloheximide (CHX). Treated plants were then incubated for 4h. Proteins were extracted at the indicated time points and analysed by western blotting using anti-HA or anti-FLAG antibodies as described above.

### Investigation of flowering time

To examine the effect of sumoylation on FLC-mediated flowering, transgenic plants carrying *35S- FLC-FLAG*_*3*_ or *35S-mFLC-FLAG*_*3*_ were generated. After selection of *FLC-FLAG*_*3*_- or *mFLC-FLAG3*-overexpressingtransgenic plants, wild-type (WT) and transgenic plants were grown in soil under long-day conditions (16h light/8h dark). Flowering time was assessed by counting the number of rosette leaves present at the time of appearance of inflorescences or was also determined by counting the days to flowering.

### Yeast two-hybrid assays

Yeast two-hybrid assay was performed using the GAL4-based two-hybrid system (Clontech). Full-length *AtSIZ1* and *IAA4* cDNAs were cloned into pGAD424 and pGBT8 (Clontech) to generate the constructs *AD-AtSIZ1*and *BD-IAA4*. The constructs were transformed into the yeast strain AH109 with the lithium acetate method. The yeast cells were grown on minimal medium (–Leu/–Trp). Transformants were plated onto minimal medium (–Leu/–Trp/–His) to test the interactions between AtSIZ1 and IAA4.

## Results

### AtSIZ1 physically interacts with FLC

**Fig. 1.**
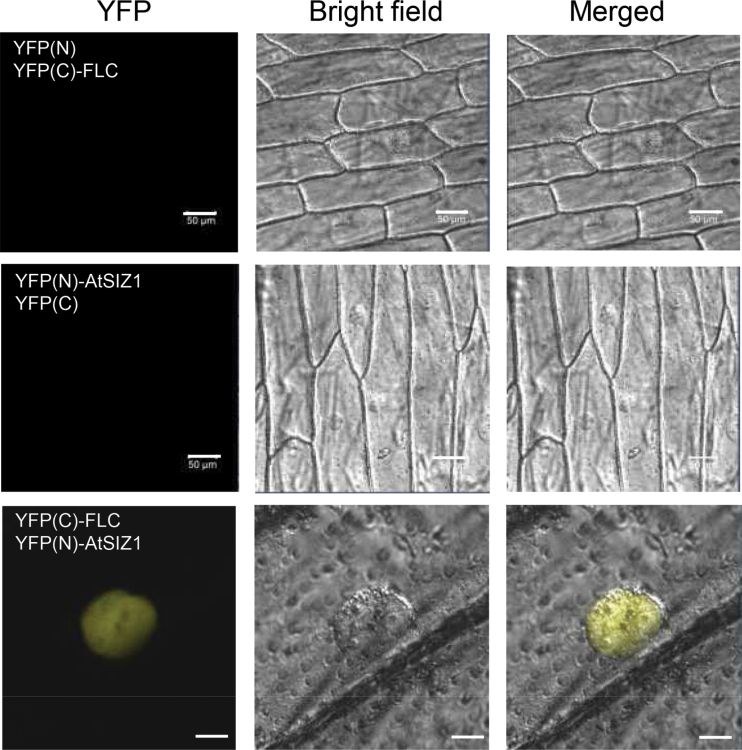
FLC interacts with AtSIZ1 *in vivo*. The interaction between AtSIZ1 and FLC was examined by a bimolecular fluorescence complementation (BiFC) assay in onion epidermal cells. AtSIZ1 and FLC cDNAs were fused with YFP at the N-terminal (N) and C-terminal (C) ends, respectively. Each combination of YFP(N)/35S–YFP(C)-FLC, 35S-YFP(N)–AtSIZ1/YFP(C), and 35S-YFP(C)–FLC/35S-YFP(N)–AtSIZ1 was introduced into onion epidermal cells by particle bombardment, and fluorescence signals were detected by confocal microscopy. Bar=50 μm.

**Fig. 2.**
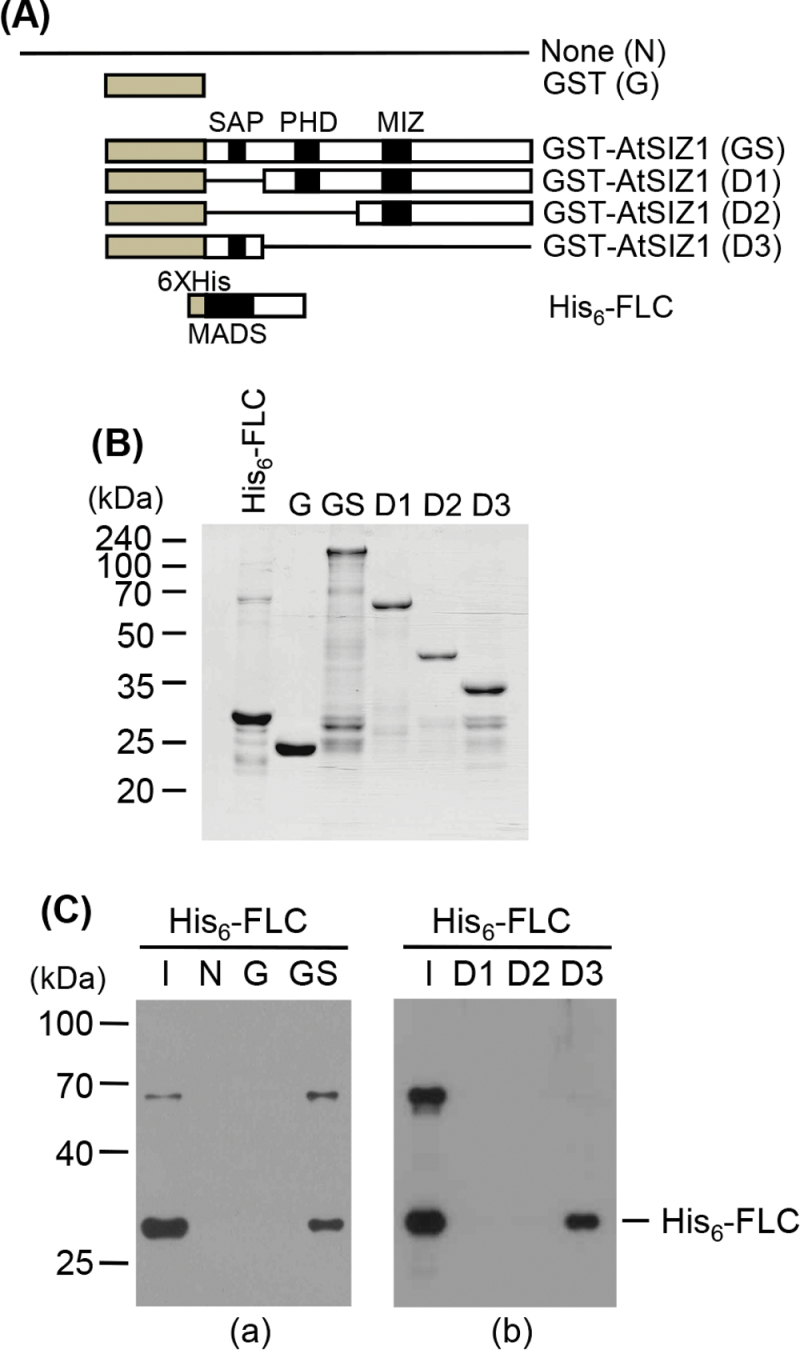
Interaction of AtSIZ1 with FLC. (A) Schematic diagram of bait [GST (G), GST–AtSIZ1 (GS), GST–AtSIZ1 (D1), GST–AtSIZ1 (D2), and GST–AtSIZ1 (D3)] and prey (His_6_-FLC) proteins. *In vitro* pull-down of FLC with AtSIZ1. (B) His_6_-FLC, full-length AtSIZ1, or its deletion mutants were overexpressed in *E. coli* and purified with Ni^2+^-NTA or glutathione affinity columns. (C) The His_6_-FLC protein was pulled down with full-length AtSIZ1 or its deletion mutant proteins, separated on 11% SDS–polyacrylamide gels, and analysed by western blotting with an anti-His antibody. I, input (His_6_-FLC).

It was recently reported that FLC directly interacts and co-localizes with the *Arabidopsis* E3 ubiquitin ligase SINAT5 in the nucleus (Park *et al.*, 2007). Since the SP-RING motif protein AtSIZ1 also localizes to the nucleus (Miura *et al.*, 2005), the possible physical interaction between AtSIZ1 and FLC was examined using a BiFC assay system. *Arabidopsis* FLC tagged with the C-terminus of EYFP and AtSIZ1 tagged with the N-terminus of EYFP were transiently expressed in onion epidermal cells. It is not known to what extent onion cells reflect the situation in *Arabidopsis* cells. Nevertheless, yellow fluorescence was detected (Fig. 1), indicating the direct interaction of these proteins *in vivo*. To confirm the interaction between FLC and AtSIZ1 in an *in vitro* system, pull-down assays were performed by overexpressing the recombinant proteins in *E. coli* and purifying them with affinity columns (Fig. 2B). Figure 2C shows that GST–AtSIZ1, but not GST alone, was able to pull down *Arabidopsis* FLC. Experiments using deletion mutants showed that the N-terminal region containing the SAP domain of AtSIZ1 [GST–AtSIZ1 (D3)] interacts with FLC (Fig. 2C). Therefore, these *in vitro* results suggest that the co-localization of FLC and AtSIZ1 in the nucleus probably reflects their direct interaction *in vivo*.

### FLC is sumoylated without AtSIZ1

**Fig. 3.**
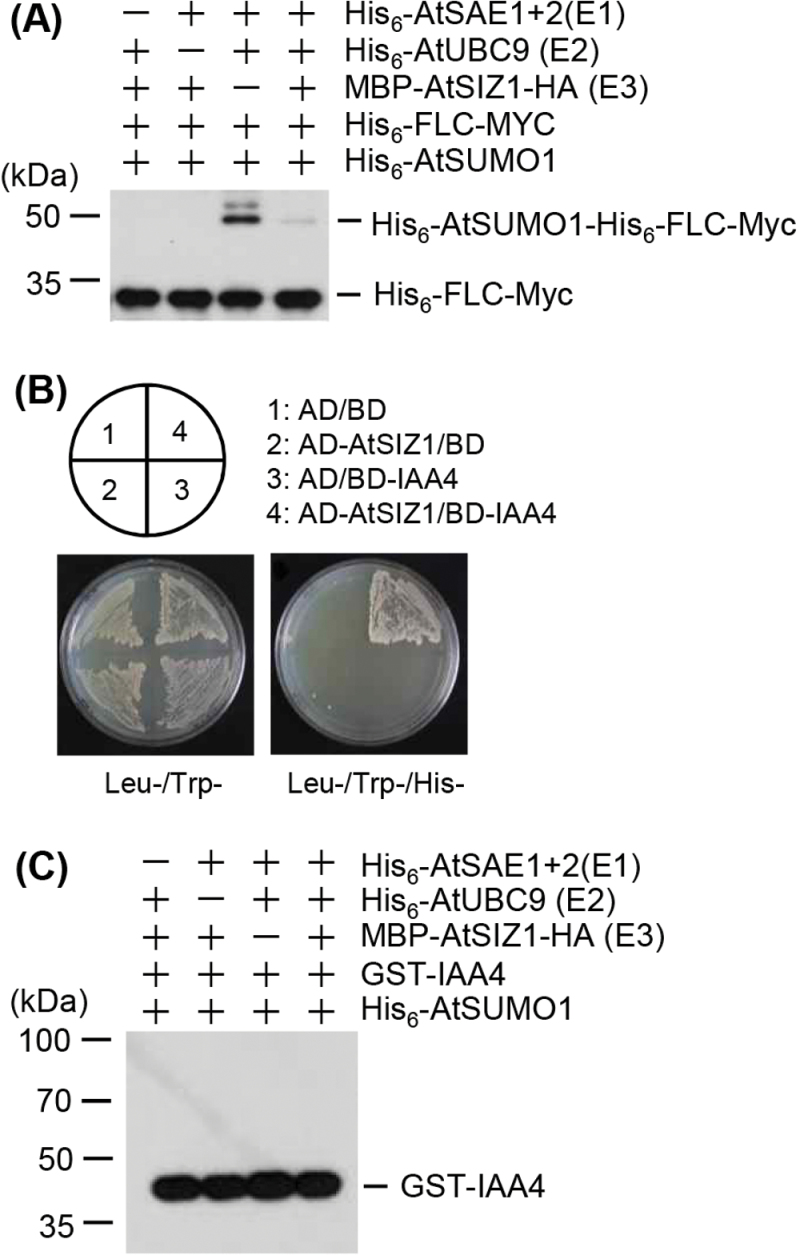
FLC is sumoylated *in vitro*. (A) *Arabidopsis* His_6_-AtSAE1b, His_6_-AtSAE2, His_6_-AtSCE1, MBP–AtSIZ1, His_6_-AtSUMO1, and His_6_-FLC-Myc were overexpressed in *E. coli* and purified with Ni^2+^-NTA, glutathione, and amylose affinity columns, respectively. Sumoylation of His_6_-FLC-Myc was assayed in the presence or absence of E1 (His_6_-AtSAE1b and His_6_-AtSAE2), E2 (His_6_-AtSCE1), E3 (MBP–AtSIZ1), and His_6_-AtSUMO1. After the reaction, sumoylated FLC was detected by western blotting with an anti-Myc antibody. GST–IAA4 was also used for the sumoylation assay as a negative control. (B) AtSIZ1 directly interacts with GST–IAA4 in yeast. Full-length AtSIZ1 and IAA4cDNAs were fused to sequences encoding the Gal4 activation domain (AD) and the Gal4 DNA-binding domain (BD) in pGAD424 and pGBT8, respectively. The constructs were transformed into yeast strain AH109. Each number indicates the yeast cells transformed with a combination of only pGAD424 and pGBT8 vectors or recombinant plasmids. Transformants were plated onto minimal medium –Leu/–Trp or –Leu/–Trp/–His and incubated for 4 d. (C) Sumoylation of GST–IAA4 was assayed using the same reaction conditions as above. After the reaction, IAA4 was detected by western blotting with an anti-GST antibody.

The direct interaction of FLC and AtSIZ1 indicated by the *in vivo* and *in vitro* results led to the hypothesis that AtSIZ1 may function as an E3 SUMO ligase for FLC. Therefore, the recombinant proteins GST–AtSIZ1-HA_3_ and His_6_-FLC-Myc were produced to determine whether AtSIZ1 is the E3 SUMO ligase for FLC. In the *in vitro* sumoylation experiments, purified His_6_-FLC-Myc was sumoylated in the presence of E1 and E2 activities (Fig. 3A). However, the sumoylation of His_6_-FLC-Myc was not induced by AtSIZ1. It was also tested whether another AtSIZ1-interacting protein, IAA4, could be sumoylated by AtSIZ1 (Fig. 3B). The result showed that IAA4 was not sumoylated under the reaction conditions employed, including the presence of E1, E2, and E3 (Fig. 3C).

### AtSIZ1 inhibits FLC sumoylation

**Fig. 4.**
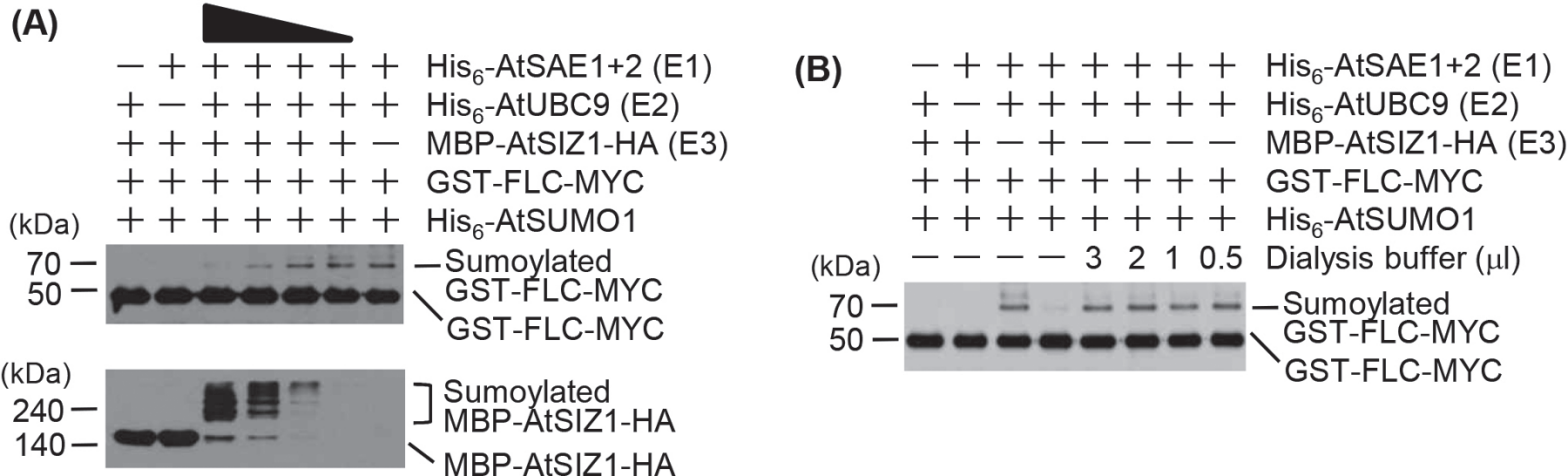
FLC sumoylation is blocked by AtSIZ1. (A) The effect of AtSIZ1 on FLC sumoylation was examined. Purified GST–AtSIZ1-HA was added to the reaction mixture at concentrations ranging from 0.1 μg to 1.0 μg. After the reaction, sumoylated FLC and AtSIZ1 were detected by western blotting with anti-Myc and anti-HA antibodies, respectively. (B) The effect of dialysis buffer on FLC sumoylation was also examined.

Despite the interaction between AtSIZ1 and FLC shown in Figs 1 and 2, the results indicate that AtSIZ1 has no E3 SUMO ligase activity for FLC (Fig. 3A). Therefore, experiments were carried out to examine whether AtSIZ1 could block or inhibit the sumoylation of FLC. The addition of increasing amounts of AtSIZ1 protein to the reaction mixture resulted in the gradual inhibition of FLC sumoylation (Fig. 4A). However, AtSIZ1 was sumoylated under the reaction conditions used here (Fig. 4A), indicating that AtSIZ1 is active and that it has self-sumoylation activity under the reaction conditions used. Since all purified proteins used in this experiment were dialysed prior to the reaction, to confirm the effect of AtSIZ1 on FLC sumoylation, an equal volume of dialysis buffer was added to the reactions; this buffer had no effect on FLC sumoylation (Fig. 4B). Therefore, these results indicate that FLC sumoylation is blocked by the AtSIZ1 protein.

### Identification of sumoylation sites on FLC

**Fig. 5.**
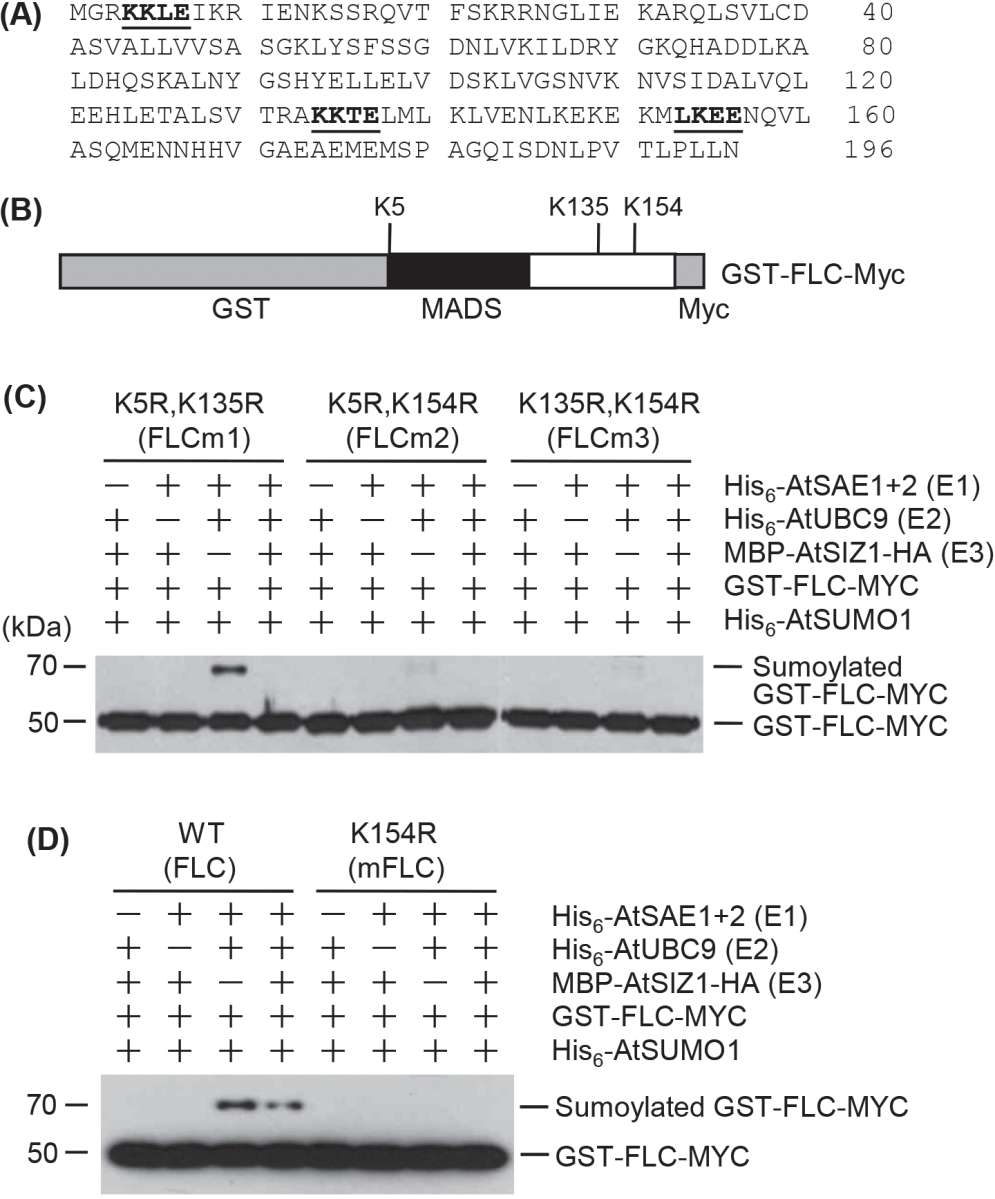
*In vitro* identification of the sumoylation site on FLC. (A) Deduced amino acid sequences of the FLC protein. Three putative sumoylation sites (ΨKXE) identified using the SUMOplot™ Analysis Program are indicated in bold type. (B) Schematic diagram of the recombinant GST–FLC-Myc protein. The MADS-box and putative sumoylation sites (K5, K135, and K154) are indicated. (C and D) *In vitro* sumoylation assays. Recombinant GST–FLC-Myc, GST–mFLC-Myc, GST–FLCm1-Myc, GST–FLCm2-Myc, and GST–FLCm3-Myc were overexpressed in *E. coli* and purified using a glutathione affinity column. The reaction mixture contained E1 (His_6_-AtSAE1b and His_6_-AtSAE2), E2 (His_6_-AtSCE1), E3 (GST–AtSIZ1), and His_6_-AtSUMO1 without (–) or with (+) a substrate protein. The mutant proteins mFLC, FLCm1, FLCm2, and FLCm3 have amino acid substitutions at residues that are predicted to be SUMO conjugation sites in FLC, as indicated. After the reaction, sumoylated FLC protein was detected by western blotting with an anti-Myc antibody.

The deduced amino acid sequences of FLC showed three putative sumoylation sites (ΨKXE) located at Lys5 (K5), Lys135 (K135), and Lys154 (K154; Fig. 5A, B). To identify the sumoylation sites on the FLC protein, single or double mutant derivatives were generated with the mutations K154R, K5R/K135R, K5R/K154R, and K135R/K154R. The proteins were overexpressed in *E. coli*, purified with glutathione affinity columns, and used for *in vitro* sumoylation assays. *In vitro* sumoylation with the double mutant proteins GST–FLCm1-Myc (K5R/K135R), GST–FLCm2-Myc (K5R/K154R), GST–FLCm3-Myc (K135R/K154R), and GST–mFLC-Myc (K154R) showed that GST–FLCm1-Myc was sumoylated, whereas GST–FLCm2-Myc, GST–FLCm3-Myc, and GST–mFLC-Myc were not (Fig. 5C). *In vitro* sumoylation assays including the single mutant protein GST–mFLC-Myc (R) showed that this protein was not modified with SUMO (Fig. 5D), indicating that K154 is the principal site of SUMO conjugation on FLC.

### FLC is stabilized by AtSIZ1

**Fig. 6.**
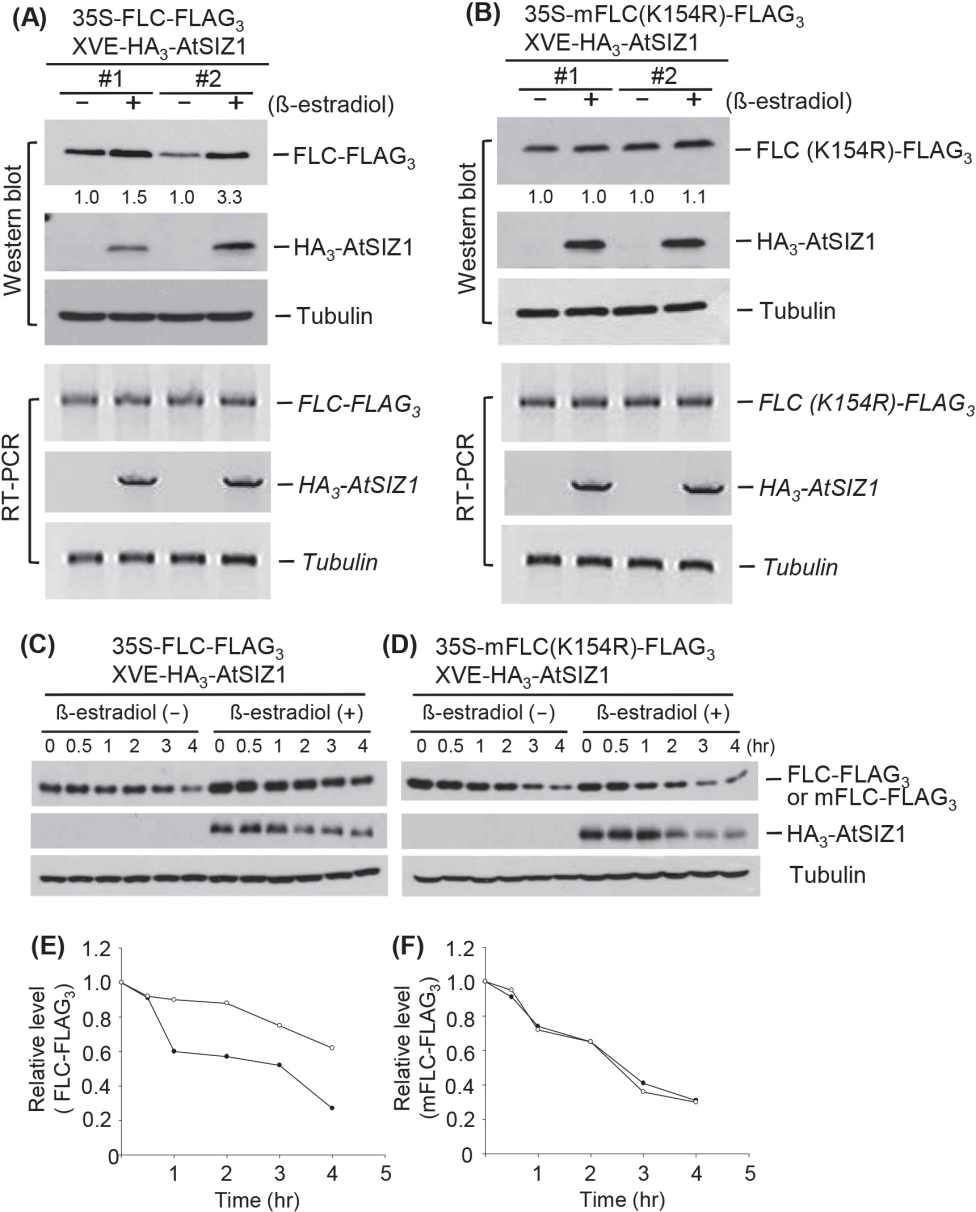
FLC is stabilized by AtSIZ1 *in vivo*. Double transgenic plants of *35S-FLC-FLAG*_*3*_ and *XVE-HA*_*3*_ *-AtSIZ1* (A) or *35S-mFLC (K154R)-FLAG*_*3*_ and *XVE-HA*_*3*_ *-AtSIZ1* (B) were incubated in liquid medium with β-oestradiol for the induction of *AtSIZ1* expression. After incubation for 15h, HA_3_-AtSIZ1, FLC-FLAG_3_, and mFLC-FLAG_3_ levels were assessed by western blotting with anti-HA or anti-FLAG antibodies. Tubulin was used as a loading control. Numbers under lanes indicate relative intensities. Protein levels were normalized to a value of 1.00 for FLC or mFLC levels in the ‘−’ inducer in both panels. RNA concentrations for *FLC-FLAG*_*3*_ and *mFLC-FLAG*_*3*_ were determined by real-time RT–PCR using a FLAG primer and a gene-specific primer. For *HA*_*3*_ *-AtSIZ1*, RNA concentration was measured by real-time RT–PCR using an HA primer and a gene-specific primer. *Tubulin* RNA was used as a loading control. To assess the degradation of FLC, double transgenic plants of *35S-FLC-FLAG*_*3*_ and *XVE-HA*_*3*_ *-AtSIZ1* (C) or *35S-mFLC (K154R)-FLAG*_*3*_and *XVE-HA*_*3*_ *-AtSIZ1* (D) were incubated in liquid medium with β-oestradiol for the induction of *AtSIZ1* expression, washed, and transferred to MS medium with 100 μM cycloheximide (CHX). At the indicated times, protein was extracted and analysed by western blotting with anti-HA or anti-FLAG antibodies. Tubulin was used as a loading control. FLC or mFLC levels during degradation were also expressed in graph form. The relative protein levels of FLC (E) or mFLC (F) were normalized to numerical values based on a value of 1.0 for the protein levels at 0h using the data shown in both C and D. Open circles indicate FLC (or mFLC) with AtSIZ1 and filled circles indicate FLC (or mFLC) without AtSIZ1.

The AtSIZ1–FLC interaction and the inhibition of FLC sumoylation by AtSIZ1 imply that the concentration of FLC may be regulated by the amount of AtSIZ1 present *in vivo*. FLC concentrations were therefore measured in transgenic plants carrying a *35S-FLC-FLAG*_*3*_ transgene and an oestradiol-inducible *XVE-HA*_*3*_ *-AtSIZ1* transgene. Induction of the expression of *AtSIZ1* increased the FLC concentrations up to 1.5- and 3.3-fold in two independent transgenic plants, respectively (Fig. 6A). However, the two independent transgenic plants carrying a *35S-mFLC-FLAG*_*3*_ transgene and an oestradiol-inducible *XVE-HA*_*3*_ *-AtSIZ1* transgene showed no changes in mFLC concentration in response to *AtSIZ1* induction (Fig. 6B). It may be possible that the transcript levels of *FLC* or *mFLC* can affect the levels of FLC and mFLC proteins in transgenic plants. Thus *FLC* and *mFLC* transcript levels were examined by real-time reverse transcription–PCR (RT–PCR) and quantitative real-time RT–PCR after induction of *AtSIZ1* in *FLC-* or *mFLC*-overexpressing double transgenic plants. The result showed that the transcript levels of *FLC* and *mFLC* were comparable under these conditions (Fig.6A, B; Supplementary Fig. S1 at *JXB* online).

The effect of AtSIZ1 on FLC decay was examined by treating the transgenic plants described above with CHX to block new protein synthesis. The results showed that the degradation of FLC was delayed in plants co-expressing *AtSIZ1* (Fig. 6C, E). However, the rate of decay of mFLC was not significantly altered by the expression of *AtSIZ1* (Fig. 6D, F).

### FLC modification by SUMO is necessary for flowering repression

**Fig. 7.**
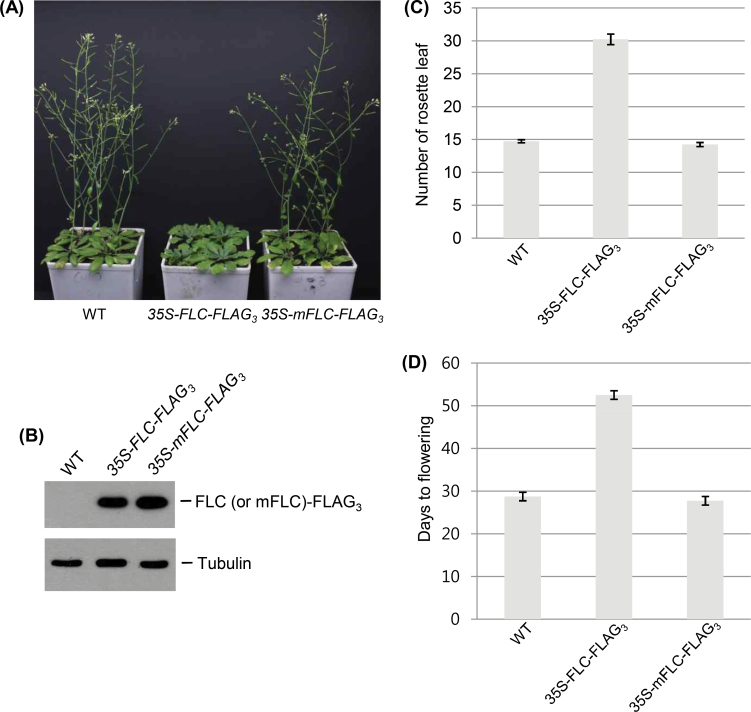
Phenotypes of FLC-overexpressing plants. (A) Vegetative growth and flowering of transgenic plants overexpressing FLC-FLAG_3_ or mFLC-FLAG_3_ were examined. (B) The protein levels of FLC-FLAG_3_ and mFLC-FLAG_3_ were examined by western blotting with anti-FLAG antibody. Tubulin was used as a loading control. (C) Flowering time in transgenic plants was examined by counting the number of rosette leaves. Significant differences were detected between WT and FLC-FLAG_3_-overexpressing plants, whereas WT and mFLC-FLAG_3_-overexpressing plants were almost identical (*P* < 0.0001, *t*-test, *n*=12). (D) The days to flowering were also determined to be identical (*P* < 0.0001, *t*-test, *n*=12). In both cases (C and D), bars indicate standard errors.

*FLC* overexpression causes late flowering, and FLC mutants are characterized by early flowering in *Arabidopsis* (Sanda and Amasino, 1996). Based on these known effects of FLC and the present sumoylation data, the effect of sumoylation on the activity of FLC as a repressor of the transition to flowering was next examined. *FLC*- and *mFLC*-overexpressing transgenic *Arabidopsis* plants were generated using *35S-FLC-FLAG*_*3*_ and *35S-mFLC-FLAG*_*3*_ constructs, respectively. After selecting homozygous lines (Supplementary Fig. S2 at *JXB* online), the recombinant protein levels of FLC-FLAG_3_ and 35S-mFLC-FLAG_3_ were first examined and then the transgenic plants were investigated for vegetative growth and flowering time (Fig. 7A, B). The relative flowering time of each transgenic plant was assessed by counting the number of rosette leaves. The number of rosette leaves in WT plants was 14.75±0.71, and that of *mFLC*-overexpressing plants was 14.63±1.16, which was comparable with that of the WT. However, in *FLC*-overexpressing plants, the number of rosette leaves was 30.50±4.68, which represented an ~2-fold increase (Fig. 7A, C). The relative flowering time of each transgenic plant was also determined by counting the days to flowering. The number of days before the appearance of inflorescences in WT plants was 28.65±1.23, and that of *mFLC*-overexpressing plants was 28.07±0.94, which was comparable with that of the WT. However, in *FLC*-overexpressing plants, the number of days before the appearance of inflorescences was 52.31±1.57, which represented an ~1.85-fold increase (Fig. 7D). As a result, the flowering time was significantly delayed in *FLC*-overexpressing *Arabidopsis* plants, while no changes were detected in *mFLC*-overexpressing plants (Fig. 7C, D). However, vegetative growth was not affected in FLC- or mFLC-overexpressing plants (Fig. 7A), suggesting that sumoylation is an important modification for the regulation of FLC function.

### Mutant FLC can interact with AtSIZ1 and FLC

**Fig. 8.**
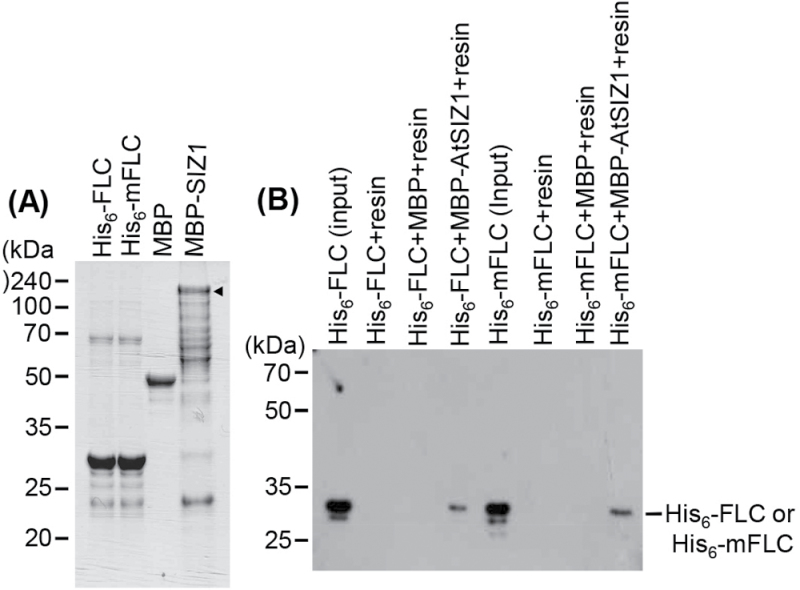
AtSIZ1 interacts with mFLC (K154R). (A) His_6_-FLC, His_6_-mFLC, and full-length MBP–AtSIZ1 were overexpressed in *E. coli* and purified with Ni^2+^-NTA or amylose affinity columns. The arrowhead indicates MBP–AtSIZ1. (B) His_6_-FLC or His_6_-mFLC proteins were pulled down with full-length MBP–AtSIZ1, separated on 11% SDS–polyacrylamide gels, and analysed by western blotting with an anti-His antibody.

**Fig. 9.**
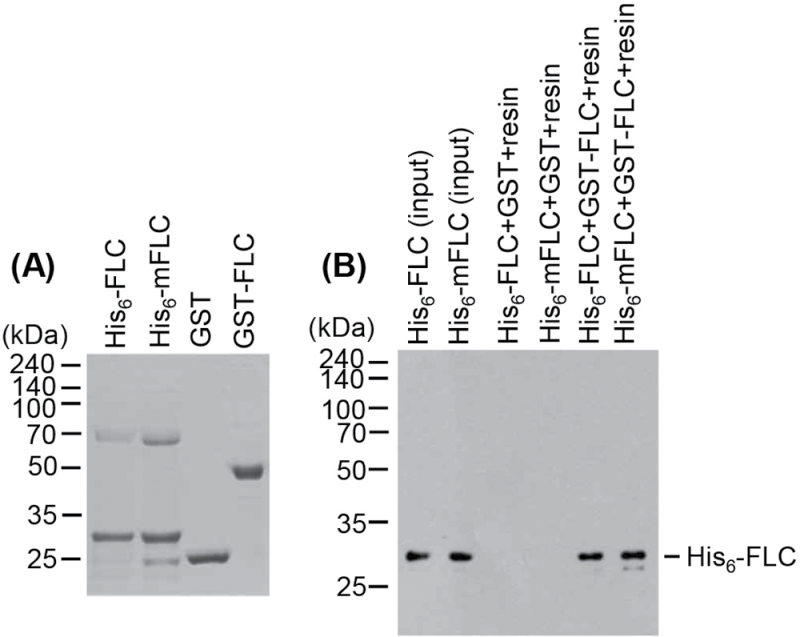
FLC can form a complex. (A) His_6_-FLC, His_6_-mFLC, GST, and GST–FLC were overexpressed in *E. coli* and purified with Ni^2+^-NTA or glutathione affinity columns. (B) His_6_-FLC or His_6_-mFLC proteins were pulled down with GST or GST–FLC proteins, separated on 11% SDS–polyacrylamide gels, transferred onto PVDF membranes, and detected by western blotting with an anti-His antibody.

The observation that AtSIZ1 stabilizes FLC but not mFLC suggests that mFLC does not interact with AtSIZ1. Therefore, the possible interaction between AtSIZ1 and mFLC was examined using an *in vitro* pull-down assay. His_6_-FLC, His_6_-mFLC, and full-length MBP–AtSIZ1 were purified with Ni^2+^-NTA or glutathione affinity columns and it was determined whether or not His_6_-FLC or His_6_-mFLC proteins could be pulled down with AtSIZ1. The results showed that AtSIZ1 interacts with both FLC and mFLC (Fig. 8). As *mFLC* overexpression had no effect on flowering time, an experiment was conducted to investigate whether mFLC can form a complex with FLC (Fig. 7). To this end, the recombinant proteins His_6_-FLC, His_6_-mFLC, GST, and GST–FLC were overexpressed in *E. coli*, these proteins were isolated with Ni^2+^-NTA or glutathione affinity columns, and whether His_6_-FLC or His_6_-mFLC proteins could be pulled down with GST or GST–FLC proteins was examined. As shown in Fig. 9, GST–FLC formed a complex with both His_6_-FLC and His_6_-mFLC.

## Discussion

In the present study, it was shown that FLC-mediated flowering repression is activated by sumoylation and that AtSIZ1 stabilizes FLC.

Eukaryotic cells express SP-RING finger proteins, SAP and Miz-finger domain (Siz) proteins, and protein inhibitor of activated STAT (PIAS) proteins (Hochstrasser, 2001). Recently, SIZ1-type proteins with a SP-RING domain were also identified in plants and were shown to be involved in diverse biological processes (Ishida *et al.*, 2012; Novatchkova *et al.*, 2012).

The function and stability of transcription factors are modulated by various post-translational modifications. The conjugation of SUMO (a protein modifier) to a target protein regulates its function and stability. FLC is modified by ubiquitin (Park *et al.*, 2007), indicating that other post-translational modifications, such as sumoylation, may play a role in the regulation of FLC activity. Experiments were therefore carried out to examine whether AtSIZ1 has E3 SUMO ligase activity for FLC. The results of pull-down and BiFC assays showed a strong interaction between FLC and AtSIZ1 (Fig. 1), and *in vitro* sumoylation assays showed that FLC is modified by SUMO (Fig. 3). However, the results showed that the attachment of SUMO to FLC occurred independently of AtSIZ1 *in vitro* (Figs 3, 4).

The covalent attachment of SUMO to a lysine residue in the target protein is generally mediated by E3 SUMO ligases. However, direct transfer from the SUMO-conjugating enzyme Ubc9 can occur through at least two ligase-independent mechanisms. First, Ubc9 can directly recognize the sumoylation motif Ψ-K-x-[D/E] (Ψ, an aliphatic branched amino acid; x, any amino acid) and conjugate the lysine residue (Bernier-Villamor *et al.*, 2002). Secondly, some SUMO substrates contain SUMO-interacting motifs (SIMs) that promote their own conjugation (Meulmeester *et al.*, 2008; Zhu *et al.*, 2008). These SIMs bind to the SUMO moiety to which Ubc9 is attached, thereby increasing its local concentration and facilitating sumoylation. The results of the present study indicate that FLC is sumoylated by one of these mechanisms in the absence of an E3 SUMO ligase.

Since FLC sumoylation was inhibited by AtSIZ1 (Fig. 4), the mechanisms underlying the binding of AtSIZ1 to FLC and its effect on FLC activity and stability were further examined. For this purpose, double transgenic *Arabidopsis* plants were generated through transformation with a *35S-FLC-FLAG*_*3*_ transgene and an oestradiol-inducible *XVE-HA*_*3*_ *-AtSIZ1* transgene to examine the effect of AtSIZ1 on the stability of FLC. *AtSIZ1* induction with oestradiol increased the concentration of FLC but not that of mFLC (Fig. 6A, B). Furthermore, *AtSIZ1* overexpression retarded the degradation of FLC, whereas that of mFLC was not affected (Fig. 6C, D). To confirm these results, the biological effect of AtSIZ1 on FLC and mFLC function and stability is also currently being investigated using double transgenic plants that constitutively overexpress AtSIZ1 and FLC or mFLC.

In any case, based on the present findings, these data suggest that AtSIZ1 stabilizes FLC through direct binding to FLC before or after FLC sumoylation *in vivo* (Supplementary Fig. S3 at *JXB* online). Furthermore, the inhibitory effect of AtSIZ1 on FLC sumoylation suggests the possible existence of another E3 SUMO ligase for FLC in *Arabidopsis* (Supplementary Fig. S3).

However, there may be many factors affecting FLC conjugation with SUMO *in vivo*. For example, *in vivo* concentrations of proteins comprising the sumoylation machinery, including *Arabidopsis* SUMO-activating enzyme E1 (SAE1+2) and conjugating enzyme E2 (AtUBC9), AtSUMO, and AtSIZ1, may differ from the concentrations of the proteins used in the *in vitro* system used here, and the expression of each of these components may vary according to developmental stage, thereby affecting FLC sumoylation. In addition, AtSIZ1 can form complexes with various proteins *in vivo* (Novatchkova *et al.*, 2012), which affects AtSIZ1 conformation and activity, and, thus, FLC sumoylation. In addition, the timing and localization of *FLC* expression can also be controlled by changes in chromatin structure through histone modifications and DNA methylation (He, 2012). FLC can form complexes with other proteins as well, which can lead to changes in FLC concentration and conformation, thereby leading to increases or decreases in the sumoylation of this protein. Therefore, the possibility that AtSIZ1 enhances FLC sumoylation as an E3 SUMO ligase *in vivo* still cannot be ruled out.

Since FLC is a central regulator of flowering, extensive research has been conducted to elucidate the mechanisms regulating FLC expression at the transcriptional and post-transcriptional levels in association with flowering time (He and Amasino, 2005; Kim *et al.*, 2005; Zhao *et al.*, 2005; Krichevsky *et al.*, 2006; Greb *et al.*, 2007; Park *et al.*, 2007; Swiezewski *et al.*, 2009; Heo and Sung, 2011). In the present study, the role of FLC in the transition to flowering was examined using the sumoylation site mutant mFLC. To characterize the function of FLC in the control of flowering time, *FLC*- or *mFLC*-overexpressing transgenic *Arabidopsis* plants were generated and their flowering time was examined by counting the number of rosette leaves. *FLC* overexpression delayed flowering, whereas *mFLC* overexpression had no notable effect on flowering time (Fig. 7A, B), indicating that sumoylation is critical for FLC to exert its floral repressor function.

The lack of an effect of *mFLC* overexpression on flowering time may have resulted from an impaired interaction of mFLC with AtSIZ1 or a defect in complex formation with FLC. However, *in vitro* pull-down analysis showed that mFLC interacted with AtSIZ1 and with FLC. From these results, several possible mechanisms explaining why *mFLC* overexpression does not affect flowering time are proposed. First, sumoylation of the FLC protein may be necessary for its activation. As mFLC cannot be modified with SUMO, this protein may not have an effect on flowering time despite its overexpression. Secondly, mFLC may inactivate endogenous FLC. Transgenic mFLC may form a complex with endogenous FLC and act in a dominant-negative form. Thus, a possible reason for the observation that flowering time in *mFLC*-overexpressing plants is comparable with that of WT plants is that the FLC level is originally low in WT plants, although this protein could be scavenged by the overexpressed mFLC through complex formation.

It is believed that if sumoylated FLC can be detected *in vivo*, it may also be possible to find an answer for why *FLC* overexpression delayed flowering, whereas *mFLC* overexpression had no effect on flowering time. However, to date, it has not been possible to detect sumolyated protein *in vivo*, perhaps due to its low level or presence at specific stages. Recently, Robertson *et al.* (2008) showed that endogenous FLC can be detected by western blot analysis with anti-FLC antibody, but the FLC band intensities were quite weak, even in C24 WT plants. It is well known that FLC protein levels are much lower in the Col background than in the C24 background. Thus, there appear to be specific challenges in detecting FLC in the Col background using antibodies. Production of a specific anti-FLC antibody which works well *in vivo* will be a solution.

DET1 (De-etiolated 1), a SINAT5-interacting partner, blocks the ubiquitination of LHY (Long Hypocotyl) by SINAT5 through direct interaction with SINAT5 (Park *et al.*, 2007). The present data show that AtSIZ1 inhibits the sumoylation of FLC through direct interaction with FLC *in vitro* (Fig. 4). However, AtSIZ1 increased the level of FLC in transgenic plants (Fig. 6A). Furthermore, the degradation of FLC was delayed in the presence of AtSIZ1 (Fig. 6C). These findings suggest that direct binding of AtSIZ1 to FLC protects the protein from degradation induced by its ubiquitination by SINAT5, as shown for DET1, which blocks the ubiquitination of LHY by SINAT5. AtSIZ1 may thus have a protective effect on FLC by antagonizing its ubiquitination (Supplementary Fig. S3 at *JXB* online).

In conclusion, the present results indicate that AtSIZ1 controls the stability of FLC by directly binding to FLC, but not through its E3 SUMO ligase activity, and that the FLC-mediated floral transition is negatively regulated by SUMO conjugation. In addition, it was shown that proteolytic turnover of flowering-associated proteins can be regulated by sumoylation. The biochemical mechanisms underlying the regulation of FLC function and stability by sumoylation were also elucidated. Together with previous findings, the data suggest that both of the post-translational modification systems, ubiquitination and sumoylation, can regulate flowering by direct modulation of FLC stability and activity.

## Supplementary data

Supplementary data are available at *JXB* online.

Figure S1. The effect of AtSIZ1 on *FLC* transcript levels.

Figure S2. Selection of FLC- and mFLC-overexpressing plants.

Figure S3. Possible regulatory modes of FLC stability.

Table S1. List of primers used for this study.

Supplementary Data

## Abbreviations:

BiFC: bimolecular fluorescence complementation
CHX: cycloheximide
DTT: dithiothreitol
EYFP: enhanced yellow fluorescent protein
GST: glutathione *S*-transferase
HA: haemagglutinin
IAA4: indoleacetic acid 4
IPTG: isopropyl-β-d-thiogalactoside
PMSF: phenylmethylsulphonyl fluoride
SUMO: small ubiquitin-related modifier
XVE: estradiol-inducible promoter.

## References

[CIT0001] Bernier-Villamor V Sampson DA Matunis MJ Lima CD 2002 Structural basis for E2-mediated SUMO conjugation revealed by a complex between ubiquitin-conjugating enzyme Ubc9 and RanGAP1. Cell 108, 345–356 11853669 10.1016/s0092-8674(02)00630-x

[CIT0002] Castro PH Tavares RM Bejarano ER Azevedo H 2012 SUMO, a heavyweight player in plant abiotic stress responses. Cellular and Molecular Life Sciences 69, 3269–8323 22903295 10.1007/s00018-012-1094-2 PMC11114757

[CIT0003] Catala R Ouyang J Abreu IA Hu Y Seo H Zhang X Chua NH 2007 The Arabidopsis E3 SUMO ligase SIZ1 regulates plant growth and drought responses. The Plant Cell 19, 2952–2966 17905899 10.1105/tpc.106.049981 PMC2048692

[CIT0004] Clough SJ Bent AF 1998 Floral dip: a simplified method for *Agrobacterium*-mediated transformation of *Arabidopsis thaliana* . The Plant Journal 16, 735–743 10069079 10.1046/j.1365-313x.1998.00343.x

[CIT0005] Colby T Matthai A Boeckelmann A Stuible HP 2006 SUMO-conjugating and SUMO-deconjugating enzymes from Arabidopsis. Plant Physiology 142, 318–332 16920872 10.1104/pp.106.085415 PMC1557612

[CIT0006] Conti L Price G O’Donnell E Schwessinger B Dominy P Sadanandom A 2008 Small ubiquitin-like modifier proteases OVERLY TOLERANT TO SALT1 and -2 regulate salt stress responses in Arabidopsis. The Plant Cell 20, 2894–2908 18849491 10.1105/tpc.108.058669 PMC2590731

[CIT0007] Elrouby N Coupland G 2010 Proteome-wide screens for small ubiquitin-like modifier (SUMO) substrates identify Arabidopsis proteins implicated in diverse biological processes. Proceedings of the National Academy of Sciences, USA 107, 17415–17420 10.1073/pnas.1005452107 PMC2951436 20855607

[CIT0008] Garcia-Dominguez M March-Diaz R Reyes JC 2008 The PHD domain of plant PIAS proteins mediates sumoylation of bromodomain GTE proteins. Journal of Biological Chemistry 283, 21469–21477 18502747 10.1074/jbc.M708176200

[CIT0009] Greb T Mylne JS Crevillen P Geraldo N An H Gendall AR Dean C 2007 The PHD finger protein VRN5 functions in the epigenetic silencing of Arabidopsis FLC. Current Biology 17, 73–78 17174094 10.1016/j.cub.2006.11.052

[CIT0010] He Y 2012 Chromatin regulation of flowering. Trends in Plant Science 17, 556–562 22658650 10.1016/j.tplants.2012.05.001

[CIT0011] He Y Amasino RM 2005 Role of chromatin modification in flowering-time control. Trends in Plant Science 10, 30–35 15642521 10.1016/j.tplants.2004.11.003

[CIT0012] Heo JB Sung S 2011 Vernalization-mediated epigenetic silencing by a long intronic noncoding RNA. Science 331, 76–79 21127216 10.1126/science.1197349

[CIT0013] Hochstrasser M 2001 SP-RING for SUMO: new functions bloom for a ubiquitin-like protein. Cell 107, 5–8 11595179 10.1016/s0092-8674(01)00519-0

[CIT0014] Hotson A Chosed R Shu H Orth K Mudgett MB 2003 *Xanthomonas* type III effector XopD targets SUMO-conjugated proteins in planta. Molecular Microbiology 50, 377–389 14617166 10.1046/j.1365-2958.2003.03730.x

[CIT0015] Ishida T Yoshimura M Miura K Sugimoto K 2012 MMS21/HPY2 and SIZ1, two Arabidopsis SUMO E3 ligases, have distinct functions in development. PLoS One 7, e46897 23056518 10.1371/journal.pone.0046897 PMC3466189

[CIT0016] Jin JB Jin YH Lee J 2008 The SUMO E3 ligase, AtSIZ1, regulates flowering by controlling a salicylic acid-mediated floral promotion pathway and through effects on FLC chromatin structure. The Plant Journal 53, 530–540 18069938 10.1111/j.1365-313X.2007.03359.x PMC2254019

[CIT0017] Kim SY He Y Jacob Y Noh YS Michaels S Amasino RM 2005 Establishment of the vernalization-responsive, winter-annual habit in Arabidopsis requires a putative histone H3 methyl transferase. The Plant Cell 17, 3301–3310 16258034 10.1105/tpc.105.034645 PMC1315370

[CIT0018] Krichevsky A Gutgarts H Kozlovsky SV Tzfira T Sutton A Sternglanz R Mandel G Citovsky V 2006 C2H2 zinc finger-SET histone methyltransferase is a plant-specific chromatin modifier. Developmental Biology 303, 259–269 17224141 10.1016/j.ydbio.2006.11.012 PMC1831845

[CIT0019] Kurepa J Walker JM Smalle J Gosink MM Davis SJ Durham TL Sung DY Vierstra RD 2003 The small ubiquitin-like modifier (SUMO) protein modification system in Arabidopsis. Accumulation of SUMO1 and -2 conjugates is increased by stress. Journal of Biological Chemistry 278, 6862–6872 12482876 10.1074/jbc.M209694200

[CIT0020] Lee J Nam J Park HC 2007 Salicylic acid-mediated innate immunity in Arabidopsis is regulated by SIZ1 SUMO E3 ligase. The Plant Journal 49, 79–90 17163880 10.1111/j.1365-313X.2006.02947.x

[CIT0021] Lois LM Lima CD Chua NH 2003 Small ubiquitin-like modifier modulates abscisic acid signaling in Arabidopsis. The Plant Cell 15, 1347–1359 12782728 10.1105/tpc.009902 PMC156371

[CIT0022] Meulmeester E Kunze M Hsiao HH Urlaub H Melchior F 2008 Mechanism and consequences for paralog-specific sumoylation of ubiquitin-specific protease 25. Molecular Cell 30, 610–669 18538659 10.1016/j.molcel.2008.03.021

[CIT0023] Michaels SD Amasino RM 1999 FLOWERING LOCUS C encodes a novel MADS domain protein that acts as a repressor of flowering. The Plant Cell 11, 949–956 10330478 10.1105/tpc.11.5.949 PMC144226

[CIT0024] Miller MJ Barrett-Wilt GA Hua Z Vierstra RD 2010 Proteomic analyses identify a diverse array of nuclear processes affected by small ubiquitin-like modifier conjugation in Arabidopsis. Proceedings of the National Academy of Sciences, USA 107, 16512–16517 10.1073/pnas.1004181107 PMC2944710 20813957

[CIT0025] Miura K Hasegawa PM 2010 Sumoylation and other ubiquitin-like posttranslational modifications in plants. Trends in Cell Biology 20, 223–232 20189809 10.1016/j.tcb.2010.01.007

[CIT0026] Miura K Jin JB Lee J Yoo CY Stirm V Miura T Ashworth EN Bressan RA Yun DJ Hasegawa PM 2007 SIZ1-mediated sumoylation of ICE1 controls CBF3/DREB1A expression and freezing tolerance in Arabidopsis. The Plant Cell 9, 1403–1414 17416732 10.1105/tpc.106.048397 PMC1913760

[CIT0027] Miura K Lee J Miura T Hasegawa PM 2010 SIZ1 controls cell growth and plant development in Arabidopsis through salicylic acid. Plant and Cell Physiology 51, 103–113 20007967 10.1093/pcp/pcp171

[CIT0028] Miura K Ohta M 2010 SIZ1, a small ubiquitin-related modifier ligase, controls cold signaling through regulation of salicylic acid accumulation. Journal of Plant Physiology 167, 555–560 19959255 10.1016/j.jplph.2009.11.003

[CIT0029] Miura K Rus A Sharkhuu A 2005 The Arabidopsis SUMO E3 ligase SIZ1 controls phosphate deficiency responses. Proceedings of the National Academy of Sciences, USA 102, 7760–7765 10.1073/pnas.0500778102 PMC1140425 15894620

[CIT0030] Murtas G Reeves PH Fu YF Bancroft I Dean C Coupland G 2003 A nuclear protease required for flowering-time regulation in Arabidopsis reduces the abundance of SMALL UBIQUITIN-RELATED MODIFIER conjugates. The Plant Cell 15, 2308–2319 14507998 10.1105/tpc.015487 PMC197297

[CIT0031] Novatchkova M Tomanov K Hofmann K Stuible HP Bachmair A 2012 Update on sumoylation: defining core components of the plant SUMO conjugation system by phylogenetic comparison. New Phytologist 195, 23–31 22799003 10.1111/j.1469-8137.2012.04135.x PMC3399776

[CIT0032] Park BS Sang WG Yeu SY Choi YD Paek NC Kim MC Song JT Seo HS 2007 Post-translational regulation of FLC is mediated by an E3 ubiquitin ligase activity of SINAT5 in Arabidopsis. Plant Science 173, 269–275

[CIT0033] Park BS Song JT Seo HS 2011 Arabidopsis nitrate reductase activity is stimulated by the E3 SUMO ligase AtSIZ1. Nature Communications 2, 400 10.1038/ncomms1408 PMC3160146 21772271

[CIT0034] Robertson M Helliwell CA Dennis ES 2008 Post-translational modifications of the endogenous and transgenic FLC protein in Arabidopsis thaliana. Plant and Cell Physiology 49, 1859–1866 18988635 10.1093/pcp/pcn167

[CIT0035] Samach A Onouchi H Gold Se E Ditta ZS Schwarz-Sommer Z Yanofsky MF Coupland G 2000 Distinct roles of CONSTANS target genes in reproductive development of Arabidopsis. Science 288, 1613–1616 10834834 10.1126/science.288.5471.1613

[CIT0036] Sanda SL Amasino RM 1996 Interaction of FLC and late-flowering mutations in *Arabidopsis thaliana* . Molecular and General Genetics 251, 69–74 8628249 10.1007/BF02174346

[CIT0037] Sheldon CC Burn JE Perez PP Metzger J Edwards JA Peacock WJ Dennis ES 1999 The FLF MADS box gene: a repressor of flowering in Arabidopsis regulated by vernalization and methylation. The Plant Cell 11, 445–458 10072403 10.1105/tpc.11.3.445 PMC144185

[CIT0038] Simpson GG Dean C 2002 Arabidopsis, the Rosetta stone of flowering time? Science 296, 285–289 11951029 10.1126/science.296.5566.285

[CIT0039] Swiezewski S Liu F Magusin A Dean C 2009 Cold-induced silencing by long antisense transcripts of an *Arabidopsis* Polycomb target. Nature 462, 799–802 20010688 10.1038/nature08618

[CIT0040] Wilkinson KA Henley JM 2010 Mechanisms, regulation and consequences of protein SUMOylation. Biochemical Journal 428, 133–145 20462400 10.1042/BJ20100158 PMC3310159

[CIT0041] Yoo CY Miura K Jin JB Lee J Park HC Salt DE Yun DJ Bressan RA Hasegawa PM 2006 SIZ1 small ubiquitin-like modifier E3 ligase facilitates basal thermotolerance in Arabidopsis independent of salicylic acid. Plant Physiology 42, 1548–1558 17041025 10.1104/pp.106.088831 PMC1676064

[CIT0042] Zhao Z Yu Y Meyer D Wu C Shen WH 2005 Prevention of early flowering by expression of FLOWERING LOCUS C requires methylation of histone H3 K36. Nature Cell Biology 7, 1256–1260 10.1038/ncb1329 16299497

[CIT0043] Zheng Y Schumaker KS Guo Y 2012 Sumoylation of transcription factor MYB30 by the small ubiquitin-like modifier E3 ligase SIZ1 mediates abscisic acid response in *Arabidopsis thaliana* . Proceedings of the National Academy of Sciences, USA 109, 12822–28227 10.1073/pnas.1202630109 PMC3411956 22814374

[CIT0044] Zhu J Zhu S Guzzo CM Ellis NA Sung KS Choi CY Matunis MJ 2008 Small ubiquitin-related modifier (SUMO) binding determines substrate recognition and paralog-selective SUMO modification. Journal of Biological Chemistry 283, 29405–29415 18708356 10.1074/jbc.M803632200 PMC2570875

