# Supplementary material for: FLC-mediated flowering repression is positively regulated by sumoylation
Source: J Exp Bot. 2013 Nov 11;65(1):339–51. doi: 10.1093/jxb/ert383 (PMC3883301; doi:10.1093/jxb/ert383)
Supplement: Supplementary Data [file supp_65_1_339__index.html]

FLC-mediated flowering repression is positively regulated by sumoylation — FLC-mediated flowering repression is positively regulated by sumoylation — Supplementary Data 

# FLC-mediated flowering repression is positively regulated by sumoylation

## Supplementary Data

Data files

**Files in this Data Supplement:**

- Supplementary Data - Supplementary Data
