# Supplementary material for: FLC-mediated flowering repression is positively regulated by sumoylation
Source: J Exp Bot. 2013 Nov 11;65(1):339–51. doi: 10.1093/jxb/ert383 (PMC3883301; doi:10.1093/jxb/ert383)
Supplement: Supplementary Data [file supp_ert383_jexbot106229_file001.pdf]

FLC-mediated flowering repression is positively regulated by sumoylation

Ga Hyun Son, Bong Soo Park, Jong Tae Song, and Hak Soo Seo

Supplementary material

Materials and methods

Quantitative real-time RT-PCR analysis

To assess the expression level of the FLC or mFLC transcripts in double transgenic plants carrying 35S-FLC-FLAG3 and XVE-3HA-SIZ1 or 35S-mFLC-FLAG3 and XVE-3HA-SIZ1, two-week-old transgenic plants were treated with β-estradiol and total RNAs were isolated from the plants. First-strand cDNA was synthesized with 5 μg of total RNA using an iScriptcDNA Synthesis Kit (Bio-Rad). An equal volume of cDNA was amplified by quantitative real-time PCR (MyiQ, Bio-Rad) according to the manufacturer's protocol. The specific primers and template cDNA were combined with 25 μL iQ SYBR Green Super Mix (Bio-Rad), and the reactions were performed under the following thermal conditions: 50°C for 2 min; 95°C for 10 min; 40 cycles of 95°C for 15 s; and 60°C for 1 min. The C_T values of target genes were normalized to the C_T value of the tubulin gene and analyzed with iCycler IQ software (Bio-Rad). All reactions were repeated three times with separate RNA samples. RT-PCR primers were designed using Primer3 (http://frodo.wi.mit.edu/cgi-bin/primer3/primer3.cgi), and their specificity was verified by cloning into the pGEM T-Easy vector (Promega) and sequencing with an ABI 3730xl DNA Analyzer (Applied Biosystems). Forward primer: 5'-tcatcatgtgggagcagaag-3' and reverse primer: 5'-tccttgtcatcgtcatcctt-3'.

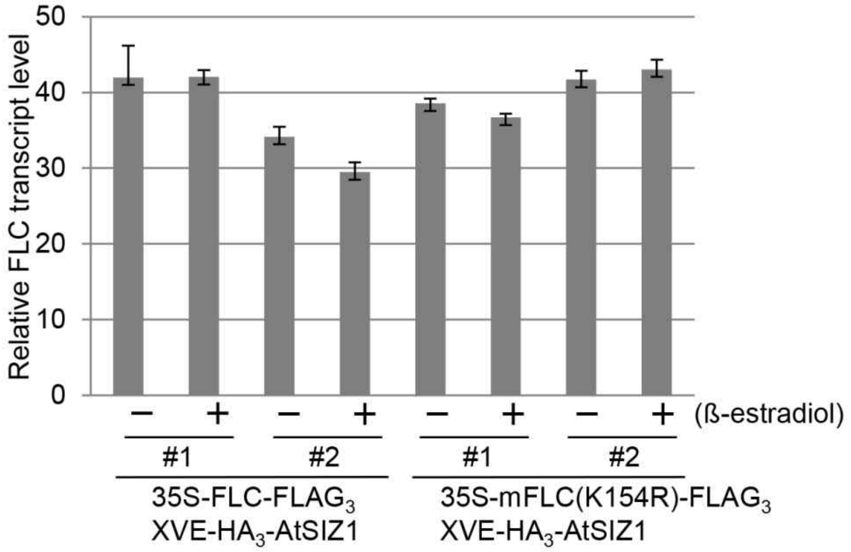

Supplementary Fig. S1. The effect of AtSIZ1 on *FLC* transcript levels. Double transgenic plants of *35S–FLC–FLAG$_3$* and *XVE–HA$_3$–AtSIZ1* or *35S–mFLC (K154R)–FLAG$_3$*and *XVE–HA$_3$–AtSIZ1* were incubated in liquid medium with β-estradiol for the induction of *AtSIZ1* expression. After incubation for 15 hours, the RNA concentrations for *FLC-FLAG$_3$* and *mFLC-FLAG$_3$* were determined by quantitative real-time RT-PCR using a FLAG primer and a gene-specific primer.

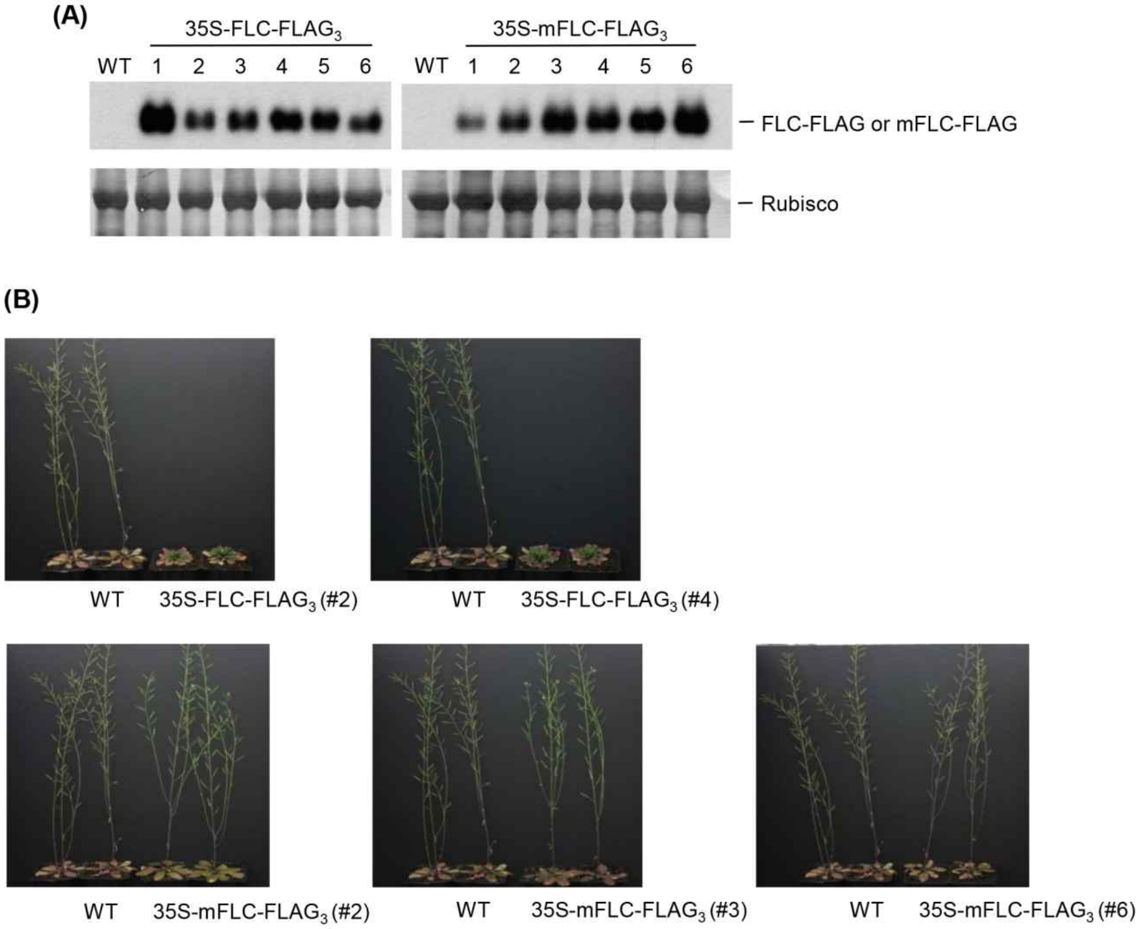

Supplementary Fig. S2. Selection of FLC- and mFLC-overexpressing plants. *Arabidopsis* was transformed with *35S-FLC-FLAG$_3$*or *35S-mFLC-FLAG$_3$*constructs. Transgenic plants overexpressing FLC-FLAG$_3$ or mFLC-FLAG$_3$ were selected and homozygous transgenic plants were chosen for further experiment. (A) The protein levels of FLC-FLAG$_3$ and mFLC-FLAG$_3$ were examined by western blotting with anti-FLAG antibody. (B) After flowering, line number 2 and 4 (of transgenic plants overexpressing FLC-FLAG$_3$) and line number 2, 3, and 6 (of transgenic plants overexpressing mFLC-FLAG$_3$) were photographed.

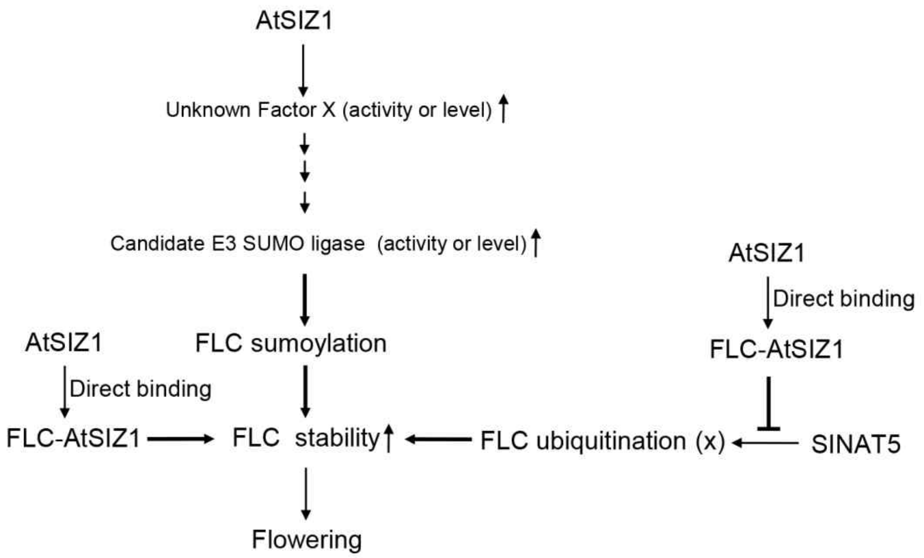

Supplementary Fig. S3. Possible regulatory modes of FLC stability. Based on sumoylation and ubiquitination data (Park *et al*., 2007), to date, three possible scenarios are suggested to explain how AtSIZ1 can stabilize FLC. Firstly, AtSIZ1 may induce the expression of an unknown factor (a type of transcription factor) or increase its activity, and the activity or level of a candidate E3 SUMO ligase for FLC would then be increased. Subsequently, this factor may sumoylate FLC, resulting in an increase in the stability and activity of FLC. Secondly, AtSIZ1 may directly bind to FLC in vivo, although it inhibits FLC sumoylationin vitro, causing FLC stabilization. Thirdly, the direct binding of AtSIZ1 with FLC may inhibit FLC ubiquitination via SINAT5 and thereby stabilize FLC.

Supplementary Table S1. List of primers used for this study

Purpose	Gene	Vector	Sequence
BiFC experiment	FLC	pDONR201	F 5′-AAAAAGCAGGCTGCATGGGAAGAAAAAAACTAGAA-3′<br>R 5′-AGAAAGCTGGGTTCTAATTAAGTAGTGGGAGAGT-3′
	AtSIZ1	pDONR201	F 5′-AAAAAGCAGGCTGCATGGATTTGGAAGCTAATTGT-3′<br>R 5′-AGAAAGCTGGTTTTACTCAGAATCCGAGTCAAT-3′
Gateway cloning	attB1		F 5′-GGGGACAAGTTTGTACAAAAAAGCAGGCT-3′
	attB2		R 5′-GGGGACCACTTTGTACAAGAAAGCTGGGT-3′
Pull down assay	FLC- Myc	pET28a, pGEX4T-1	F 5′-TCTACGAATTCATGGGAAGAAAAAAACTA-3'<br>R 5′-TCTACCTCGAGTTATTCATTCAAGTCCTCTTCAGAAATGAGCTTT<br>TGCTCCATATTAAGTAGTGGGAGAGT-3
	AtSIZ1-HA	pMALc2X	F 5′-TCTACGAATTCATGGATTTGGAAGCTAAT-3′<br>R 5′-TCTACTCTAGATTAAAGACTAGCATAATCTGGAACATCATAAGG<br>ATACATCTCAGAATCCGAGTCAAT-3′
	AtSIZ1(D1)<br>:△PHD,△MIZ	pGEX4T-1	F 5′-TCTACGGATTCATGGATTTGGAAGCTAATTGTAAG-3′<br>R 5′-TCTACCTCGAGTTACTTAACTTTCAGATTACTGGTATC-3′
	AtSIZ1 (D2)<br>:△SAP	pGEX4T-1	F 5′-TCTACGAATTCATGAGTTCAGATACCAGTAATCTGAAA-3′<br>R 5′-TCTACGTCGACTTAGCTACCATCAGGTGCATGCCACTG-3′
	AtSIZ1(D3)<br>:△SAP,PHD	pGEX4T-1	F 5′-TCTACGAATTCATGAGACTTGTGAAGCGCAGGACTCTA-3′<br>R 5′-TCTACGTCGACTTAGCTACCATCAGGTGCATGCCACTG-3′
*In vitro*sumoylation assay	AtSUMO1-GG	pET28a	F 5′-TCTACGAATTCATGTCTGCAAACCAGGAGGAAGAC-3′<br>R 5′-TCTACCTCGAGTTAGCCACCAGTCTGATGGAGCAT-3′
Site directed mutagenesis	FLC (K5R)-Myc	pGEX4T-1	F 5′-TCTAC GAATTCATGGGAAGAAAAAGGCTAGAAAT-3′<br>R 5′-TCTACCTCGAGTTATTCATTCAAGTCCTCTTCAGAAATGAGCTTTTG<br>CTCCATATTAAGTAGTGGGAGAGT-3′
	FLC (K135R)- Myc	pGEX4T-1	F 5′-TCTACGAATTCATGGGAAGAAAAAAACTA-3'<br>R 5′-GAGAAAATGCTGAGAGAAGAGAACC-3′<br><br>F 5′-CTAGAGCCAAGAGGACCGAACT-3′<br>R 5′-TCTACCTCGAGTTATTCATTCAAGTCCTCTTCAGAAATGAGCTTTTG<br>CTCCATATTAAGTAGTGGGAGAGT-3′
	FLC (K154R)- Myc	pGEX4T-1	F 5′-TCTACGAATTCATGGGAAGAAAAAAACTA-3'<br>R 5′-GGTTCTCTTCTCTCAGCATTTTC-3′<br><br>F 5′-GAGAAAATGCTGAGAGAAGAGAACC-3′<br>R 5′-TCTACCTCGAGTTATTCATTCAAGTCCTCTTCAGAAATGAGCTTTTG<br>CTCCATATTAAGTAGTGGGAGAGT-3′
Production of transgenic plants	FLC-FLAG$_3$	pBA002	F 5′-TCTACCTCGAGATGGGAAGAAAAAAACTAGAAATC-3′<br>R 5′-TCTACGAGCTCTCACTTGTCATCGTCATCCTTGTAGTCCTTGTCA<br>TCGTCATCCTTGTAGTCCTTGTCATCGTCATCCTTGTAGTCCATA<br>T-3′
	HA$_3$-AtSIZ1	pBA002	F 5′-TCTACCCCGGGATGGATTTGGAAGCTAAT-3'<br>R 5′-TCTACACTAGTTTACTCAGAATCCGAGTC
